# Splice Variants of the Forkhead Box Protein AFX Exhibit Dominant Negative Activity and Inhibit AFXα-Mediated Tumor Cell Apoptosis

**DOI:** 10.1371/journal.pone.0002743

**Published:** 2008-07-23

**Authors:** Eun Jig Lee, Jeong Mo Kim, Mi Kyung Lee, J. Larry Jameson

**Affiliations:** 1 Endocrinology, Yonsei University College of Medicine, Seoul, Korea; 2 Division of Endocrinology, Metabolism, and Molecular Medicine, Feinberg School of Medicine, Northwestern University, Chicago, Illinois, United States of America; 3 Pathology, National Health Insurance Corporation, Ilsan Hospital, Koyang, Korea; National Institute on Aging, United States of America

## Abstract

**Background:**

Loss-of-function in the apoptosis-inducing genes is known to facilitate tumorigenesis. AFX (FOXO4), a member of forkhead transcription factors functions as a tumor suppressor and has 2 isoforms, AFXα (505 a.a.) and AFXζ (450 a.a.). In human cancer cells, we identified an N-terminally deleted form of AFXα (α198-505), translated from a downstream start and 2 short N-terminal AFX proteins (90, and 101 a.a.) produced by aberrant splicing.

**Methods and Findings:**

We investigated the expression and role of these AFX variants. Cell transduction study revealed that short N-terminal AFX proteins were not stable. Though α(198-505) protein expression was detected in the cytoplasm and nucleus, α(198-505) expressing cells did not show a nucleocytoplasmic shuttling mediated by PI3 kinase signaling. Whereas, we observed this shuttling in cells expressing either AFXα or AFXζ protein. AFXζ and α(198-505) lost the ability to transactivate BCL6 or suppress cyclin D2 gene expression. These variants did not induce cancer cell death whereas AFXα resulted in apoptosis. We found that AFXζ and α(198-505) suppress the AFXα stimulation of BCL6 promoter in a dose dependent manner, indicating dominant negative activity. These variants also inhibited AFXα induction of apoptosis.

**Conclusions:**

Loss of function by aberrant splicing and the dominant negative activity of AFX variants may provide a mechanism for enhanced survival of neoplastic cells.

## Introduction

AFX (FOXO4) is a member of the class O (FOXO) subfamily of forkhead transcription factors that includes the functionally related proteins FOXO1 (FKHR), FOXO3a (FKHRL1), and FOXO4 [1], [2]. FOXO transcription factors have important roles in metabolism, cellular proliferation, and apoptosis. Overproduction of FOXOs induces either cell cycle arrest or apoptosis. When relieved of FOXO activity, cells re-enter the cell cycle and start to proliferate [3]. A role for FOXO members in tumorigenesis was initially suggested by the observation that FOXO members are involved chromosomal translocations in certain types of tumors [4], [5]. The PAX3-FKHR fusion product has been shown to transform cells in culture [6]. However, translocations involving FOXO proteins might also result in loss-of-function of a FOXO allele.

AFX is known to have two isoforms, AFXα (505 amino acids) and AFXζ (450 amino acids) [7]. AFXζ is a splice variant that encodes a shorter protein lacking amino acids 58-112, including the first 16 amino acids of the forkhead domain. AFXα is ubiquitously expressed, whereas AFXζ is expressed predominantly in liver, kidney, pancreas, heart, and placenta. AFXζ transcripts are not detected in ovary, testis, brain, prostate, colon, and leukocyte. The different tissue distributions AFXα and AFXζ suggest distinct transcriptional regulation and actions on a different subset of target genes.

In this report, we describe novel spliced forms of the AFX transcript in human cancer cells and their effect on tumor apoptosis and growth.

## Results

### AFX splicing variants

**Figure 1:**
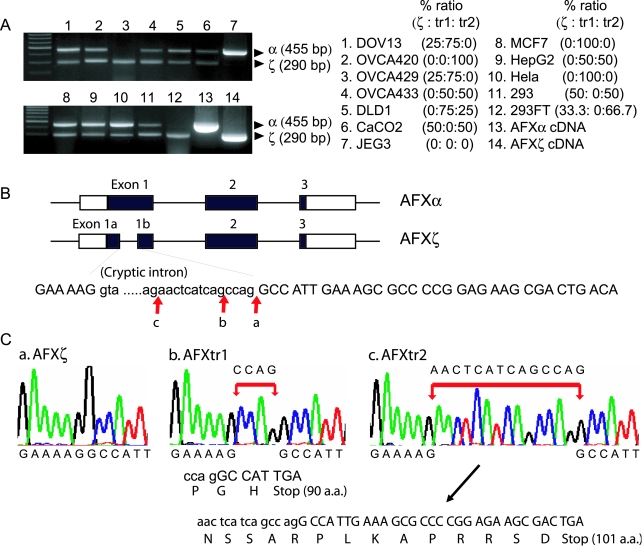
AFX splicing variants. (A) RT-PCR of total RNA samples from human cancer cell lines. The upper band (445 bp) represents AFXα and the lower band (290 bp) contains AFXζ. Lower bands were cloned and sequenced. Percent ratio of AFXζ, AFXtr1, and AFXtr2 was calculated. 1-12, Sequencing results of cloned lower bands from each cell line (α, AFXα; ζ, AFXζ; tr1, AFXtr1; and tr2, AFXtr2); 13, AFXα cDNA; and 14, AFXζ cDNA. (B, C) Schematic representation of AFXα and AFXζ, and AFX splicing variants by aberrant splicing. The splice acceptance occurred aberrantly at 4 (b) and 14 (c) bases before splice acceptor site (a) of AFXζ isoform. This aberrant splicing results in 4 and 14 base insertions, leading to a shift of reading frame and producing premature stop codon. Splicing variants with 4 and 14 bases insertion have 90 (AFXtr1) and 101 (AFXtr2) amino acids (a.a.), respectively.

PCR amplification of reverse-transcribed cDNA samples from human cancer cell lines was performed using a primer set that anneals to both AFXα and AFXζ cDNAs. The upper band (445 bp) corresponds to AFXα and lower band (290 bp) contains AFXζ. There was a slight difference in the size of AFXζ bands among the cell lines, prompting us to consider possible splicing variants (Fig. 1A). Direct sequencing of lower bands identified the AFXζ isoform, as well as overlapping and mixed sequences near splice acceptor site of AFXζ isoform (not shown). The lower bands were cloned and sequenced to investigate these sequences further. Two novel splicing variants were identified, in which the splice acceptance occurred aberrantly at 4 and 14 bases before splice acceptor site of the AFXζ isoform (Fig. 1B, C). This aberrant splicing results in either a 4 or 14 base insertion, leading to a shift of the reading frame and producing premature stop codon. Splicing variants with the 4 or 14 base insertion encode predicted proteins of 90 (AFXtr1) or 101 (AFXtr2) amino acids, respectively (Fig. 1C). A variety of tumor cells showed different patterns of AFX transcripts (Fig. 1A). Either AFXtr1 or AFXtr2 were detected in most tumor cells. AFXζ was detected in DOV13, OVCA429, CaCO2, HEK293, and 293FT cells. AFXα expression was absent in OVCA429 and 293FT cells. JEG3 cells express only AFXα. Percent ratio of AFXζ, AFXtr1, and AFXtr2 was different each other according to tumor cells (Fig. 1A).

**Figure 2:**
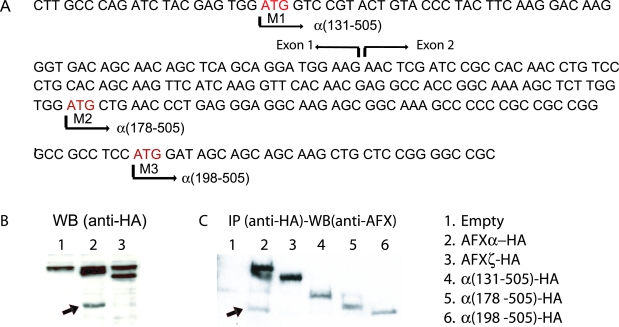
Identification of N-terminally deleted AFX protein, α(198-505). (A) Three start codons (M1, M2, and M3) are present in exon 1b and 2. M3 is the methionine codon located at the end of forkhead domain, which contains a consensus Kozak sequence for initiation of translation. (B) Western blot analysis. We detected an additional band (arrow) using anti-HA antibody from the lysate of 293-FT cells transfected with AFXα-HA (C-terminal tagged), suggesting a presence of N-terminally deleted AFX protein. (C) Immunoprecipitation analysis. To identify truncated protein, three N-terminally deleted AFX constructs tagged with C-terminal-HA epitope {α(131-505)HA, α(178-505)HA, and α(198-505)HA} were transfected. Western blotting with anti-AFX antibody (C-terminal specific) after immunoprecipitation with anti-HA antibody of transfected cell lysates revealed α(198-505) protein (arrow).

We became interested in the N-terminally truncated AFXα proteins because AFXtr1 and AFXtr2 are prematurely terminated in exon 1, and three potential alternative start codons (M1, M2, and M3) are present in exon 1b and 2 (Fig. 2A). M3 is the methionine codon located at the end of forkhead domain. It contains a consensus Kozak sequence for initiation of translation and thus potentially encodes an N-terminally deleted AFXα. Western blot analyses using an anti-HA antibody from the lysate of 293FT cells transfected with C-terminal hemaglutinin (HA) epitope-tagged AFXα detected an additional band, suggesting a presence of N-terminally deleted AFX protein (Fig. 2B, arrow). In order to identify this product, we generated three N-terminally deleted AFX constructs tagged with C-terminal-HA epitope {α(131-505)-HA, α(178-505)-HA, and α(198-505)-HA}. Western blotting with anti-AFX antibody (C-terminal specific) after immunoprecipitation with anti-HA antibody of transfected cell lysates confirmed the presence of the α(198-505) protein (Fig. 2C, arrow).

### Expression and subcellular localization of AFX variants

**Figure 3:**
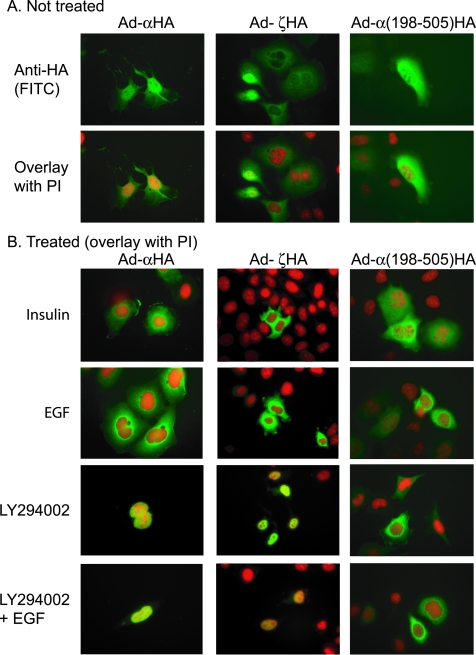
Expression and subcellular localization of AFX variants. HeLa cells were seeded on fibronectin coated cover glass and infected with adenoviral vectors carrying C-terminal HA-epitope tagged AFXs. Immunofluorescence staining using anti-HA antibody was performed. (A) Not treated. (B) Treated with insulin, EGF, LY294002, and LY294002 followed by EGF. α, AFXα; and ζ, AFXζ.

Cellular protein processing and localization of AFX variants was examined by immunofluorescence (IF) staining. JEG3 cells were transfected with the constructs carrying the N- or C-terminal HA epitope in-frame with AFXα-HA, AFXζ-HA, or α(198-505)-HA. Immunofluorescence positivity was detected in about 5–8% of JEG3 cells. No protein expression was detected in cells transfected with HA-AFXtr1 and HA-AFXtr2 (Data not shown). To achieve high levels of gene expression, we generated adenoviral vectors carrying these constructs. After determining the dose of adenoviral vectors required to achieve 95–100% expression of the control β-galactosidase gene in HeLa cells, protein expression was still not detected for vectors containing HA-AFXtr1 or HA-AFXtr2 (data not shown), whereas 90–95% of cells expressed AFXα-HA, AFXζ-HA, or α(198-505)-HA in either cytoplasms and nuclei (Fig. 3A). These results indicate that the short aminoterminal-AFX proteins are not appropriately processed or stable in the cells, although the product from the alternate translation start site α(198-505) is produced.

The effect of the cell signaling on subcellular localization was assessed by adding insulin, EGF, or LY294002 (a PI3 kinase inhibitor). Insulin and EGF treatment induced the excursions of AFXα and AFXζ to cytoplasms and LY294002 pretreatment prevented these excursions, indicating that nucleocytoplasmic shuttling of AFXα or AFXζ protein is occurred and regulated by PI3 kinase signaling pathway. Whereas α(198-505) expressing cells did not show this shuttling (Fig. 3B). This defect may be due to the fact that α(198-505) has lost two binding sites for 14-3-3 protein and half of the nuclear localization sequence (182-211 amino acids) [8], [9], [10], [11].

### AFX variants fail to transactivate the BCL6 gene or suppress the cyclin D2 gene

**Figure 4:**
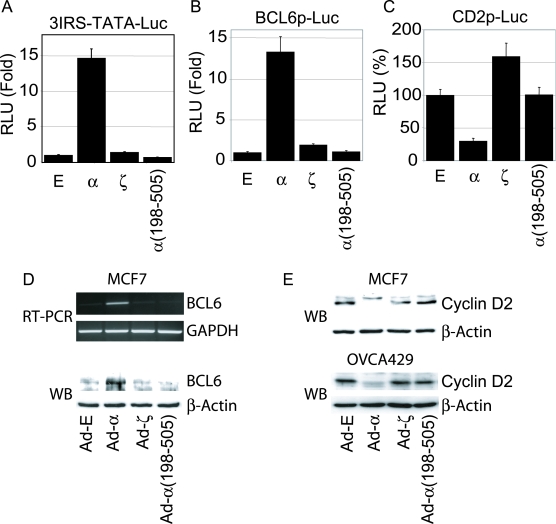
AFX variants do not transactivate the BCL6 gene or suppress the cyclin D2 gene. (A, B, C) Transcriptional activity of AFXζ and α(198-505) compared to AFXα using a luciferase reporter system. 293-FT cells were co-transfected with AFX expression vectors and luciferase reporter constructs, 3IRS-TATA-Luc (A); BCL6p-Luc (B); and CD2p-Luc (C). Luciferase values were normalized by protein measurements. Results were averaged from 3 independent experiments and are plotted as means±standard deviations for quadruplicated wells. (D) RT-PCR and Western blot analysis of BCL6 expression in MCF7 cells infected with Ad-E, Ad-α, Ad-ζ or Ad-α(198-505). (E). Western blot analysis of cyclin D2 expression in MCF7 and OVCA429 cells infected with Ad-E, Ad-α, Ad-ζ or Ad-α(198-505).

The transcriptional activity of AFXζ and α(198-505) was compared to AFXα using a luciferase reporter system. The amounts of plasmid vectors transfected were carefully optimized to minimize nonspecific effects [12] and luciferase values were normalized by protein measurements. AFXζ and α(198-505) did not stimulate 3IRS-TATA-Luc, whereas AFXα activated this reporter about 15-fold (Fig. 4A). Two representative target genes, BCL6 and cyclin D2, were also examined. BCL6 is a transcriptional repressor of BCL-X_L_, an antiapoptotic protein, and the AFX protein is thought to induce activates apoptosis by stimulation of BCL6 [13]. The cell cycle inhibition by AFX protein involves down-regulation of cyclin D [14]. In 293FT cells, AFXζ and α(198-505) did not stimulate BCL6p-Luc or suppress CD2p-Luc, whereas AFXα stimulated BCL6p-Luc 13-fold and suppressed CD2p-Luc by 75% (Fig. 4B, C). RT-PCR and Western blot analyses were used to investigate the effect of the AFX variants on the expression of BCL6 or cyclin D2 in cells infected with adenoviral vectors carrying AFXα, AFXζ, or α(198-505). Whereas AFXα induced BCL6 expression in MCF7 cells, Ad-ζ or Ad-α(198-505) did not increase either BCL6 mRNA or protein (Fig. 4D). Ad-α suppressed cyclin D2 expression, however Ad-ζ or Ad-α(198-505) lost the ability to suppress its expression in MCF7 or OVCA429 cells (Fig. 4E). These results indicate that the AFX variants are loss-of-function mutants.

### AFX variants have lost the ability to suppress tumor cell growth

**Figure 5:**
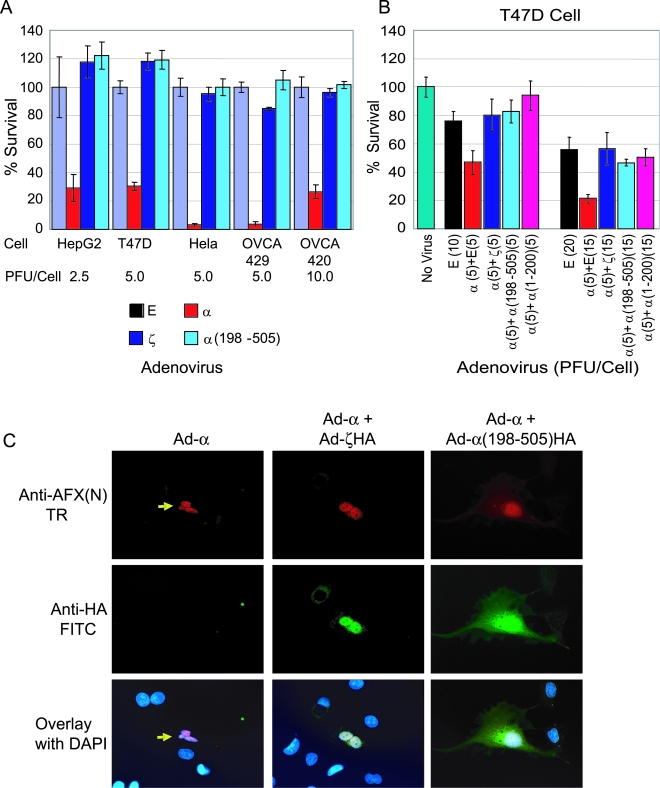
AFX variants do not suppress tumor cell growth. (A) Cell survival after transduction of AFXα, AFXζ, and α(198-505). HepG2, T47D, HeLa, OVCA429, and OVCA420 cells were infected with adenoviral vectors from 2.5 to 10 PFU/cell and cell viability was assessed using the MTS assay at day 6. The amount of adenoviral vectors for each cell line was optimized to achieve 95–100% expression levels. Percent cell survival was calculated compared to Ad-E infection. (B) AFXζ and α(198-505) inhibits AFXα induced growth suppression. We used an adenoviral vector {Ad-α(1-200)} containing AFXα(1-200), a C-terminal truncation mutant that has dominant negative activity. Four days after co-infection of adenoviral vectors, cell viability was assayed. Results were averaged from 3 independent experiments and are plotted as means±standard deviations for quadruplicated wells. (C) Prevention of Ad-α induced apoptosis by Ad-ζ and Ad-α(198-505). Double immunofluorescent staining was performed to identify cells infected with two viruses. HeLa cells infected with Ad-α showed apoptotic nuclei (arrow), whereas HeLa cells co-infected with either Ad-ζ or Ad-α(198-505) did not show apoptosis. Anti-AFX(N), N-terminal specific antibody against AFX; E, Ad-E, α; Ad-α; ζ, Ad-ζ ; ζ-HA, Ad-ζ-HA; and α(198-505), Ad-α(198-505).

HepG2, T47D, HeLa, OVCA429, and OVCA420 cells were infected with adenoviral vectors (from 2.5 to 10 PFU/cell ) expressing AFXα, AFXζ, or α(198-505). Cell viability was assessed using the MTS assay at day 6 and the percent cell survival was calculated compared to Ad-Empty (Ad-E) infection. Infection of Ad-α suppressed 70–95% cell growth, whereas Ad-ζ or Ad-α(198-505) did not suppress cell growth (Fig. 5A), indicating that the AFX variants have lost the ability to suppress tumor cell growth.

### AFX variants inhibited AFXα induction of cell death by a dominant negative effect on the BCL6 gene

We examined whether AFX variants might inhibit AFXα induction of apoptosis through dominant negative activity. An additional adenoviral vector {Ad-α(1-200)} were constructed containing AFXα(1-200), a C-terminal truncation mutant that contains a functional DNA-binding domain, but lacks a transactivation domain. A similar FOXO1(1-255) construct has been shown to have dominant negative activity [15], [16]. Ad-α(1-200) infection alone did not induce cell death in HepG2, T47D, HeLa, OVCA429, and OVCA420 cells (data not shown). Co-infection of Ad-α with equal (5 PFU/cell) or higher (15 PFU/cell) amounts of Ad-E, Ad-ζ, Ad-α(198-505), or Ad-α(1-200) in T47D cells were performed to investigate the effect of AFX variants on AFXα induction of apoptosis. Because high amounts of adenoviral vector alone induce cell death, control infections were performed using Ad-E, an MOI (10 and 20 PFU/cell) that was equal to total amount of co-infected adenoviral vectors. Co-infection of Ad-α and Ad-E induced 30–40% more cell death compared to corresponding amounts of Ad-E infection. Co-infection of Ad-ζ, Ad-α(198-505), or Ad-α(1-200) prevented AFXα induction of cell death (Fig. 5B), suggesting dominant negative activity of AFX variants. Cellular examination was also performed using double immunofluorescent. HeLa cells expressing nucleic AFXα alone showed apoptotic nuclei, whereas HeLa cells co-expressing AFXα with AFXζ or α(198-505) did not show apoptosis. Of note, AFXα was co-localized with AFXζ or α(198-505) in nuclear speckles of these cells (Fig. 5C).

**Figure 6:**
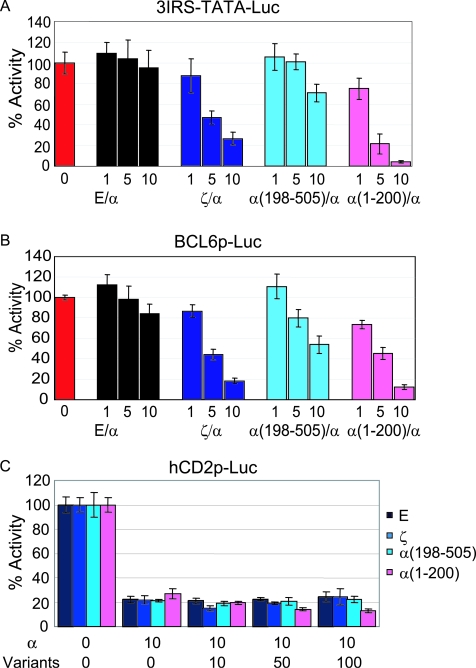
AFX variants exert dominant negative activity on the BCL6 gene promoter. (A, B, C) Co-transfection of AFXα with equal or increasing amounts of the AFX variants was performed in the context of the luciferase reporters, 3IRS-TATA-Luc (A), BCL6p-Luc (B) and CD2p-Luc (C). The α(1-200) construct was used as a known dominant negative mutant. Results were averaged from 3 independent experiments and are plotted as means±standard deviations for quadruplicated well. E, Empty; α, AFXα; and ζ, AFXζ.

The mechanism of dominant negative activity of AFX variants was assessed further by examining effects on transcriptional regulation of 3IRS-TATA-Luc, BCL6p-Luc, and CD2p-Luc. Co-transfection of AFXα with equal or increasing amounts of the AFX variants was preformed in the context of these luciferase reporters. Co-transfection with the AFX variants reduced the AFXα-induced transactivation of 3IRS-TATA-Luc and BCL6p-Luc in a dose-dependent manner (Fig. 6A, B). The reduction by AFXζ was comparable to that of α(1-200). However, the AFX variants did not reverse AFXα suppression of CD2p-Luc activity (Fig. 6C). These results suggest that the inhibition of AFXα-induced apoptosis is mediated through dominant negative activity of AFX variants on AFXα in BCL6 gene expression.

## Discussion

In this report, we identified spliced forms of AFX transcripts in multiple human cancer cell lines. Short aminoterminal AFX proteins (AFXtr1 and AFXtr2) produced by aberrant splicing were not stable, suggesting AFX inactivation by aberrant splicing. However, alternative splicing and translation produced AFXζ and α(198-505), respectively. AFXζ and α(198-505) lost the ability to transactivate BCL6 or to suppress cyclin D2 gene expression. Although inactive as individual transcription factors, AFXζ and α(198-505) exert dominant negative activity on AFXα stimulation of BCL6 gene. These variants also lost the ability to induce apoptosis but they inhibited AFXα induced apoptosis, presumably through dominant negative activity on the BCL6 gene. Inactivation of AFX by aberrant splicing and the dominant negative function of the AFX splicing variants could therefore provide a growth advantage during cancer progression.

It has been suggested that AFXα and AFXζ may antagonized each other because of their distinct transcriptional activity for different target genes. For example, AFXζ stimulates PEPCK, G6Pase promoters, whereas AFXα does not activate these promoters [7]. We also observed distinct transcriptional regulatory function for AFXα and AFXζ. The CD2 promoter was weakly stimulated by AFXζ and repressed by AFXα (Fig. 4C). A novel finding in this study is the dominant negative function of AFXζ or α(198-505) on AFXα regulation of the BCL6 promoter. A number of AFXα binding sites have been demonstrated in BCL6 promoter [13]. One AFXα binding site was also competed by a known forkhead-binding site (IRS) derived from the IGFBP-1 promoter [13]. Consistent with this, we also observed dominant negative activity by the AFX variants on AFXα stimulation of 3xIRS promoter construct. These results indicate that the dominant negative activity by AFX variants may be mediated through IRS (GCAAAACAA AC TTATTTTGAA).

The induction of BCL6 accounts for part of the apoptotic mechanism mediated by AFXα. FOXO dependent expression of IGFBP-1 [17], FasL [18], [19] and Bim [20]–[23] have also been shown to promote apoptosis. Direct FOXO binding activity has been demonstrated in the FasL promoter (GTAAATAAATA) and the Bim promoter (GTAAACAC). It is possible that the AFX variants may also influence AFXα activation of these genes.

Of note, the AFX variants did not reverse AFXα suppression of the CD2 promoter. FOXO factors have been shown to elevate p27KIP1 expression and induce cell cycle arrest [24], [25]. FOXO3a and AFX have also been shown to inhibit the cell cycle through downregulation of cyclin D by a p27KIP1-independent mechanism [2], [14], [26]. Chromatin immunopreciptation (ChIP) using the cyclin D1 [27] and cyclin D2 promoters [28] demonstrated FOXO binding on the cyclin D promoters. However, a recent study suggested that transcriptional repression of D-type cyclin may not involve direct binding of FOXO factors to cyclin D1 or D2 promoters [26]. These results suggest that transcription repressors activated by AFXα might play a role in downregulation of cyclin D.

An important question is whether there is a causal relationship between AFX splicing variants and cancer. For this to be feasible, the alternative splicing products would need to be expressed at significant levels compared with the normally spliced product [29]. We compared the pattern of AFX transcripts in two different cell lines (HEK 293 and 293FT) originated from same cells. By RT-PCR there was a decrease of the AFXα transcript in 293FT cells, whereas HEK293 cells still possessed it (Fig. 1A). HEK293 cells are transformed by the adenovirus E1 gene and 293FT cells were additionally transformed by simian virus (SV) 40 large tumor antigen. Loss of the AFXα transcript and the appearance of the AFX variants could contribute to the higher proliferation of 293FT cells relative to HEK293 cells (data not shown). However, the mechanism described in our study remains to be determined in primary tumors.

Defects in mRNA splicing occur frequently in human cancer cells [29] but the mechanisms leading to splicing defects in cancer are poorly understood. A point mutation in the genomic splice site or regulatory elements have been shown in selected splicing defects [30]–[32]. However, DNA sequencing of the intronic and exonic portions of AFX gene did not reveal mutations. Variations in the composition, concentration, localization, and activity of transacting regulatory factors may also modulate splice-site recognition and usage [33], [34]. Activation of the oncogenic signal pathway also stimulates aberrant splicing [35]. It is of interest to better understand how splicing regulatory factors in cancer cells might affect AFX aberrant splicing and thereby cell survival.

## Methods

### Cells and culture

Cell lines for ovarian cancer (DOV13, OVCA420, OVCA429, and OVCA433), colon cancer (DLD1, CaCO2), breast cancer (MCF7, T47D), choriocarcinoma (JEG3), hepatoma (HepG2), cervical cancer (HeLa), and transformed human embryonic kidney cells (HEK293, and 293FT) were cultured in DMEM/F12 supplemented with 10% fetal bovine serum (FBS), 100 units/ml penicillin, and 100 μg/ml streptomycin. All cells were maintained at 37°C with 5% CO2.

### RT-PCR and sequence analysis

Total RNA was extracted from each cells using TRIZOL reagent (Invitrogen, Carlsbad, CA) as described by the manufacturer. RNA (20 μg) was treated with DNAse-I (Promega, Madison, WI) for 30 min at room temperature. Random hexamers and AMV reverse transcriptase (RT) were used to synthesize cDNA using 10 μg of DNAse-I treated RNA. A portion (1/40) of the cDNA solution was used for amplification of AFX. Cycle conditions were: 2 min hot start at 96°C, followed by 35 cycles of 1 min at 94°C, 45 sec at 56°C, followed by 1 min at 72°C, and a final extension at 72°C for 5 minutes. An aliquot (50%) of each PCR reaction was resolved by electrophoresis on 1.8% agarose gels and DNA products were visualized with ethidium bromide. Oligonucleotides used for PCR amplification include forward 5′-ACG TAT GGA TCC GGG GAA TG-3′ and reverse 5′-TCC ATC CTG CTG AGC TGT-3′. PCR product give two bands; AFXα (445 bp) and AFXζ (290 bp). The lower AFXζ bands were excised and cloned into p-TOPO vector (Invitrogen). To identify splicing variants, 20 positive clones carrying the PCR segment from each cell line were sequenced using an automated DNA sequencer.

### Plasmids and transfections

The cDNAs for AFXα, AFXζ, AFXα(198-505), AFXα(1-200), AFXtr1, and AFXtr2 were amplified using KOD DNA polymerase (Novagen, San Diego CA) and the RT product of total RNAs from cancer cells and cloned into pCR®-Blunt (Invitrogen). After verification of sequencing, each cDNA was subcloned into pCDNA3. Fusion constructs were created by incorporating an N- or C-terminal influenza virus HA epitope (YPYDVPDYA) in-frame with the AFXs in the pCDNA3 vectors.

In a preliminary transfection study using the pGL3 basic vector (Promega, Madision WI), we observed that AFXα stimulated luciferase activity about 60-fold. We found 2 IRS-A (CAAAACAA) sequences in the synthetic polyA (spA) region and removed them (NotI and KpnI). After confirmation that neither AFXα or AFXζ stimulated spA deleted pGL3basic vector, the sequences containing 3×insulin response sequences (IRSs, G**CAAAACAA** AC **TTATTTTG**AA) [36] and TATA box, the human BCL6 promoter (−785 +55) [13], and the human cyclin D2 promoter (1302 bps) [14] were inserted into multiple cloning sites to generate 3IRS-TATA-Luc, BCL6p-Luc, and CD2p-luc, respectively.

Transfections of 293FT cells with 500 ng of luciferase constructs and 10 ng of each AFX construct were performed using the calcium phosphate method. Forty eight hrs later, cells were harvested 48h later and luciferase activity was assayed and normalized as described before [12]. Results were averaged from 3 independent experiments and are plotted as means±standard deviations for quadruplicated wells.

### Adenoviral infection and cell proliferation assay

Recombinant adenoviral vectors {Ad-α, Ad-αHA, Ad-ζ, Ad-ζHA, Ad-α(198-505), Ad-α(198-505)HA, and Ad-α(1-200)} carrying each of the AFX cDNAs, with or without HA epitope, were amplified, purified, titrated as described previously [37]. Cytotoxicity was assessed using a nonradioactive cell proliferation assay according to the manufacturer's protocol (Cell Titer 96 Aqueous Non-Radioactive Cell Proliferation Assay, Promega). The day after plating 3×10^3^ cells per well in 96-well plates, adenoviral vectors {Ad-α, Ad-ζ, and Ad-α(198-505)} were infected at various MOIs (2.5-10 plaque forming unit/cell). Empty adenoviral vector (Ad-E) was also infected as a control. To achieve 100% cellular expression of adenoviral transgene, MOIs for each cell line were determined by infection of an adenovirus carrying β-galactosidase [37]. Fresh medium (DMEM/F12 supplemented with 2.5% heat inactivated FBS) was added 8 h after infection and every 2 days thereafter. Cell viability was assayed 6 days after infection. Percent cell survival was quantitated relative to Ad-E infected or uninfected cells.

### Immunofluorescence

Transfected or infected cells were reseeded on cover glasses precoated with fibronectin. Twenty four hrs later, cells were fixed in cold methanol for 10 min and permeabilized in 0.4% Triton-X/PBS for 10 min. After washing with TBS/0.05% Tween, rat monoclonal anti-HA high affinity antibody (Clone 3F10, 2.5 μg/ml, Roche Diagnostic Co, Manm, Germany) was incubated for 2 hrs. Visualization was performed using anti-rat Ig-biotin, F(ab')2 fragment (5 μg/ml, Jackson Immunoresearch, city) and streptavidin-Texas Red (1∶100, Vector Laboratories Inc., Burlingame, CA). Cell images were analyzed using a Zeiss microscope (Axioskop, Carl Zeiss Inc., Oberkochen, Germany). For N-terminal specific antibody against AFX (Goat, AFX-N, Santa Cruz Biotechnology, Inc. Santa Cruz CA), biotinylated anti-goat antibody (1∶300, Vector lab) was used. Each antibody was optimized and titrated to minimize nonspecific activity.

### Immunoprecipitation and Western blot analysis

Immunoprecipitation and Western blot analysis using lysates from cells were performed as described previously [16], [38]. Primary antibodies used were anti-HA high affinity antibody (Roche), anti-AFX antibody (C-terminal specific, A-8975, Sigma- Aldrich, Inc. Saint Louis MO), anti-BCL6 (N-3, Santa Cruz Biotechnology) and anti-cyclin D2 (M-20, Santa Cruz Biotechnology).
